# Next-Generation Sequencing of Lung Cancer *EGFR* Exons 18-21 Allows Effective Molecular Diagnosis of Small Routine Samples (Cytology and Biopsy)

**DOI:** 10.1371/journal.pone.0083607

**Published:** 2013-12-23

**Authors:** Dario de Biase, Michela Visani, Umberto Malapelle, Francesca Simonato, Valentina Cesari, Claudio Bellevicine, Annalisa Pession, Giancarlo Troncone, Ambrogio Fassina, Giovanni Tallini

**Affiliations:** 1 Department of Medicine (DIMES) – Anatomic Pathology Unit, Bellaria Hospital, University of Bologna, Bologna, Italy; 2 Department of Pharmacology and Biotechnology (FaBiT), University of Bologna, Bologna, Italy; 3 Department of Public Health, University of Naples Federico II, Naples, Italy; 4 Department of Medicine (DIMED) – Surgical Pathology & Cytopathology Unit, University of Padua, Padua, Italy; National Taiwan University, Taiwan

## Abstract

Selection of lung cancer patients for therapy with tyrosine kinase inhibitors directed at EGFR requires the identification of specific *EGFR* mutations. In most patients with advanced, inoperable lung carcinoma limited tumor samples often represent the only material available for both histologic typing and molecular analysis. We defined a next generation sequencing protocol targeted to *EGFR* exons 18-21 suitable for the routine diagnosis of such clinical samples. The protocol was validated in an unselected series of 80 small biopsies (n=14) and cytology (n=66) specimens representative of the material ordinarily submitted for diagnostic evaluation to three referral medical centers in Italy. Specimens were systematically evaluated for tumor cell number and proportion relative to non-neoplastic cells. They were analyzed in batches of 100-150 amplicons per run, reaching an analytical sensitivity of 1% and obtaining an adequate number of reads, to cover all exons on all samples analyzed. Next generation sequencing was compared with Sanger sequencing. The latter identified 15 *EGFR* mutations in 14/80 cases (17.5%) but did not detected mutations when the proportion of neoplastic cells was below 40%. Next generation sequencing identified 31 *EGFR* mutations in 24/80 cases (30.0%). Mutations were detected with a proportion of neoplastic cells as low as 5%. All mutations identified by the Sanger method were confirmed. In 6 cases next generation sequencing identified exon 19 deletions or the L858R mutation not seen after Sanger sequencing, allowing the patient to be treated with tyrosine kinase inhibitors. In one additional case the R831H mutation associated with treatment resistance was identified in an *EGFR* wild type tumor after Sanger sequencing. Next generation sequencing is robust, cost-effective and greatly improves the detection of *EGFR* mutations. Its use should be promoted for the clinical diagnosis of mutations in specimens with unfavorable tumor cell content.

## Introduction

Lung carcinoma often presents at advanced stage and is the leading cause of cancer-related death in developed countries (http://seer.cancer.gov/statfacts/html/lungb.html#survival). The discovery in 2004 that activating somatic mutations in the tyrosine kinase *EGFR* gene make the tumor sensitive to tyrosine kinase inhibitors (TKIs) treatment has represented one of the most significant breakthrough in the field of molecular oncology in the past decade [1,2]. Randomized clinical trials have shown patient responses to the TKIs Erlotinib (Tarceva, OSI Pharmaceutical) or Gefitinib (Iressa, Astrazeneca) as first-line treatment in approximately two thirds of patients with *EGFR* mutated tumors with rates far superior to those obtained with conventional platinum-based chemotherapy [3-9]. 

*EGFR* mutations have become “critical” biomarkers to appropriately select patients for TKIs treatment, and guidelines for molecular diagnosis have been outlined by professional organizations both in Europe and in the United States [10,11]. Most - 80-90% - of the *EGFR* mutations are either small exon 19 deletions or the L858R mutation in exon 21, but other TKIs sensitive *EGFR* mutations can occur in exons 12, 19, 20, 21. Mutations associated with TKIs resistance, like the T790M in exon 20, can also develop in small tumor cell sublclones and need to be identified [1,2,8,12-23].

*EGFR* mutated tumors are typically adenocarcinomas, where mutations can be identified in approximately a quarter of cases, and in a higher proportion of tumors from Asian patients. 

Adenocarcinomas are now regarded as the most common lung carcinoma subtype, constituting approximately 40% of all non-small cell lung cancers (NSCLC) [24] and molecular analysis of *EGFR* exons 18, 19, 20, 21 is recommended in all adenocarcinoma or lung tumors with an adenocarcinoma component [10]. Thus, the pathologic evaluation of a lung carcinoma now requires both an accurate subtyping by histological and immunohistochemical studies as well as the determination of the *EGFR* mutational status to select patients for TKIs therapy. This in depth evaluation obviously requires adequate amounts of tumor tissue of good quality, like those obtained from lung resections [25].

Unfortunately 60% of NSCLC are high stage locally advanced and/or inoperable tumors that have already metastasized to distant sites by the time they are detected (http://seer.cancer.gov/statfacts/html/lungb.html#survival). Thus, in patients with such tumors very limited samples - small biopsies or cytology specimens - are usually the only material available for histologic typing and for molecular analysis [26]. In these samples the issue of specimen purity i.e. the proportion of lesional material to the “contaminating” benign or non-lesional cells is often critical [27,28]. Sanger sequencing, the most widely used method for mutation detection does not have enough analytical sensitivity to reliably identify mutations in samples with a low proportion of tumor cells. It can give false negative results if the percentage of neoplastic cells is below a general threshold of 50% that corresponds to 25% mutated alleles, assuming the mutation is heterozygous and that the *EGFR* chromosomal site 7p12 is dysomic [29]. Therefore alternative methods – each with its own advantages and disadvantages – have been proposed to detect *EGFR* mutations with the goal of achieving higher sensitivity. Many are currently used for molecular analysis of routine samples [30]. These include High Resolution Melting (HRM) [31], Restriction Fragment Length Polymorphism (RFLP) [32], mutant allele–specific PCR [33], Peptide Nucleic Acid Locked PCR Clamping (PNA-PCR) [34,35], pyrosequencying [36], and immunohistochemistry with specific EGFR antibodies that detect the L858R mutation and exon 19 deletion [37,38]. Some mutation-specific methods like the Scorpion ARMS (TheraScreen EGFR29 mutation kits from QIAGEN Manchester [formerly DxS], Manchester, England) [39] reach a very high sensitivity (~1%) but underestimate not pre-designed mutations and require a rather significant amount of DNA, not always available in limited samples [40,41].

The development of next generation sequencing (NGS) methods - also known as massive parallel sequencing since they allow the parallel analysis of a very large number of DNA molecules - has represented one of the more significant technical advances in molecular biology [42]. These methods have become available since 2005 and are producing remarkable breakthroughs in oncology, including the definition of the entire DNA sequence of common types of human cancers [43]. 

This is a multicentric study to evaluate the application of NGS to the molecular diagnosis of *EGFR* mutations in limited samples of NSCLC, small biopsies and cytology specimens. Instead of analyzing a few cases for a large number of genes, we chose to target parallel sequencing to the *EGFR* mutation “hot spots”. The focus was on *EGFR* analysis because, as mentioned above, *EGFR* mutations often need to be identified in DNA extracted from specimens that are both problematic for molecular diagnosis and at the same time are very crucial to decide patient treatment. NGS sequencing results were compared with those of Sanger sequencing, the method currently in use for the routine molecular analysis in the three Italian partner centers in Bologna, Padova and Naples, that participated to the study.

We reasoned that in spite of its greater complexity compared with Sanger sequencing (or other alternative methods) NGS offers several advantages - high analytical sensitivity, screening of the entire nucleotide sequence of the target region, semiquantitative evaluation of the mutated allele, analysis of many samples in a single run (high throughput) - that make it ideal for the study of lung carcinoma. Our results indicate that NGS can be effectively applied to meet the needs of routine DNA analysis, and that it represents a practical alternative to other methods currently used to detect *EGFR* mutations.

## Materials and Methods

### Ethics Statement

Since *EGFR* mutational analysis is part of the routine diagnostic workup of patients with NSCLC the need for ethic committee’s approval was not necessary for this study, in accordance with medical ethical guidelines of the Azienda Unità Sanitaria Locale di Bologna, Azienda Universitaria Policlinico Università degli Studi di Napoli Federico II, Azienda Universitaria Policlinico Università degli Studi di Padova and in accordance with general authorisation to process personal data for scientific research purposes from “The Italian Data Protection Authority” (http://www.garanteprivacy.it/web/guest/home/docweb/-/docweb-display/export/2485392). Accordingly to these guidelines, a comprehensive written informed consent was signed for the procedures (fine needle aspiration, biopsies and surgical resections) that produced the tissue samples and for their diagnostic workup. All information regarding the human material was managed using anonymous numerical codes. Clinical data and follow up information were not used for this study. All samples were handled in compliance with the Helsinki declaration (http://www.wma.net/en/30publications/10policies/b3/).

### Case selection and collection of tumor material for molecular analysis

Eighty samples of NSCLC were randomly selected from patients that underwent diagnostic workup at the sections of Anatomic Pathology in the Bellaria Hospital-University of Bologna and Maggiore Hospital in Bologna, and in the University Hospitals of Naples and Padova. All patients had a clinical indication for *EGFR* mutation testing for advanced lung carcinoma. Tumor cells for molecular analysis were obtained from cytology slides in 66 cases and from formalin-fixed paraffin embedded (FFPE) tissue sections in 14 biopsy specimens. Both cytology and biopsy specimens were routinely processed and diagnoses rendered according to standard criteria.

All cases were processed for DNA extraction after pathologic review ensured the presence of at least one hundred neoplastic cells. The proportion of neoplastic cells/total number of cells (i.e. tumor cell enrichment) was estimated on all cases. The total number of neoplastic cells present in the sample processed for molecular analysis was also evaluated. It was estimated as follows: in a given sample neoplastic cells were counted in five one square millimeter fields (1 mm^2^) and the mean of these five values calculated; the mean was then multiplied for the total area (in millimeters) of the specimen – cytology smear or histology section of the biopsy – selected for molecular analysis.

For cytology samples cells were scraped from the area selected for the analysis after cover-slip removal by immersion of the slide in xylene. For FFPE material, six 10 μm thick sections were cut from each selected block, followed by one Hematoxylin and Eosin stain (H&E) control slide. The tumor area was marked on the control slide and tumor material was manually dissected under microscopic guidance from the corresponding 10 μm sections using a sterile blade. Twenty additional DNA samples - previously characterized for the *EGFR* mutational status - were utilized to evaluate the reproducibility of 454 parallel sequencing (see below).

### DNA extraction

For Sanger sequencing DNA was extracted according to standard procedures as previously reported [44-46]. To ensure maximal yield of DNA for NGS two distinct protocols were followed for cytology and biopsy specimens. DNA was extracted from cytology samples using MasterPure DNA Purification Kit (Epicentre, Madison, WI, USA) according to the manufacturer’s instruction. For samples obtained from biopsies DNA was extracted with the High Pure PCR Template Preparation Kit (Roche Diagnostics, Mannheim, Germany) following the manufacturer’s protocol.

### Sequencing

Eighty samples were tested for *EGFR* (exon 18, 19, 20 and 21) using Sanger and/or Next Generation sequencing. Sanger sequencing was performed at the Molecular Pathology facilities of the Anatomic Pathology sections of the Bellaria Hospital-University of Bologna (33 cases), of the University Hospital of Naples (29 cases) and of Padova (18 cases). Next Generation sequencing of all cases was performed in parallel using a 454 GS-Junior Next Generation sequencer at the Molecular Pathology facility of the Bellaria Hospital-University of Bologna, Anatomic Pathology section, starting from routinely processed material originally selected from the Anatomic Pathology section of the Bellaria Hospital and from the submitting institutions in Padova and Naples. Negative controls and no template DNA controls were included in all runs.

#### Sanger sequencing

PCR reactions were performed using the FastStartTaq DNA polymerase kit (Roche Applied Science, Mannheim, Germany), starting from 15–50 ng of DNA. Primers used for PCR are described in Table 1. PCR products were checked on 2.5% agarose gel and amplicons purified using Agencourt Ampure XP beads (Beckman Coulter, Inc., Fullerton, CA, U.S.A.). Sequencing was carried out according to standard procedures using the GenomeLab DTCS Kit (Beckman Coulter, Inc., Fullerton, CA, U.S.A.) and a CEQ2000 XL automatic DNA sequencer (Beckman Coulter, Inc., Fullerton, CA, U.S.A.) in the Bellaria Hospital, using the BigDye Terminator kit (version 3.1; Life Technologies) and run on the ABI 3730 analyzer (Life Technologies) in the Naples University Hospital and in the Padova University Hospital.

**Table 1:** Primers used for amplification of EGFR exons 18-21.

***EGFR* Exon**	**Sanger sequencing 5’-3’**	**NGS 5’-3’^*a*^**	**Lenght (bp)**
Ex18 Fw	CATgTCTggCACTgCTTTCC	ggCTgAggTgACCCTTgTC	184
Ex18 Rv	AgggACCTTACCTTATACACC	gCCTgTgCCAgggACCTTAC	
Ex19 Fw	AgCATgTggCACCATCTCAC	AgCATgTggCACCATCTCAC	182
Ex19 Rv	ATgAgAAAAggTgggCCTgA	CCCACACAgCAAAgCAgAAA	
Ex20 Fw	AgCCACACTgACgTgCCTCT	AgCCACACTgACgTgCCTCT	208
Ex20 Rv	CCTTATCTCCCCTCCCCgTA	TgCACACACCAgTTgAgCAg	
Ex21 Fw	TgCAgAgCTTCTTCCCATgA	CCTCACAgCAgggTCTTCTC	196
Ex21 Rv	gCATgTgTTAAACAATACAgC	CCACCTCCTTACTTTgCCTCCT	

^a^ Primers for 454 next generation sequencing are linked to a sequence of 10 nucleotides (MID – Multiple Identifier), 4 nucleotide of “junction” and 25 nucleotide of “universal sequence”, according to manufacturer’s instruction (not shown in the Table). Ex, Exon; Fw, Forward; Rv, Reverse.

#### 454: Next Generation Sequencing

Sequence analysis of *EGFR* exon 18, 19, 20 and 21 was performed with the 454 GS-Junior Next Generation sequencer (Roche Diagnostics, Mannheim, Germany), according to established protocols (http://www.454.com/). 

Briefly, these include the following steps: PCR amplification of the target sequence, purification of the amplified fragments, emulsion PCR, and recovery of the emulsion PCR products that are then loaded on the Titanium PicoTiterPlate (Roche Diagnostics) of the 454 GS Junior instrument for massively parallel pyrosequencing. Primers for the initial amplification run are modified with universal tail sequences and multiple identifiers (MID) nucleotides (Integrated DNA Technologies Inc, www.idtdna.com). A specific couple of MID sequences identifies univocally each sample (target sequence).

For *EGFR* mutation analysis we defined the following workflow format, using the primers shown in Table 1 to analyze exons 18-19-20-21. Approximately 10 ng of genomic DNA were amplified for each exon. All PCR reactions were performed using a FastStart High Fidelity Taq Polymerase (Roche Diagnostic, Mannheim, Germany). In each sequencing run 100 to 120 different target sequences were analyzed for parallel pyrosequencing. This allowed us to study 25-30 cases per run, since all four *EGFR* exons were evaluated on all patients, with a putative number of approximately 700 reads per target, according to manufacturers’ specifications (http://www.454.com/).

**Figure 1:**
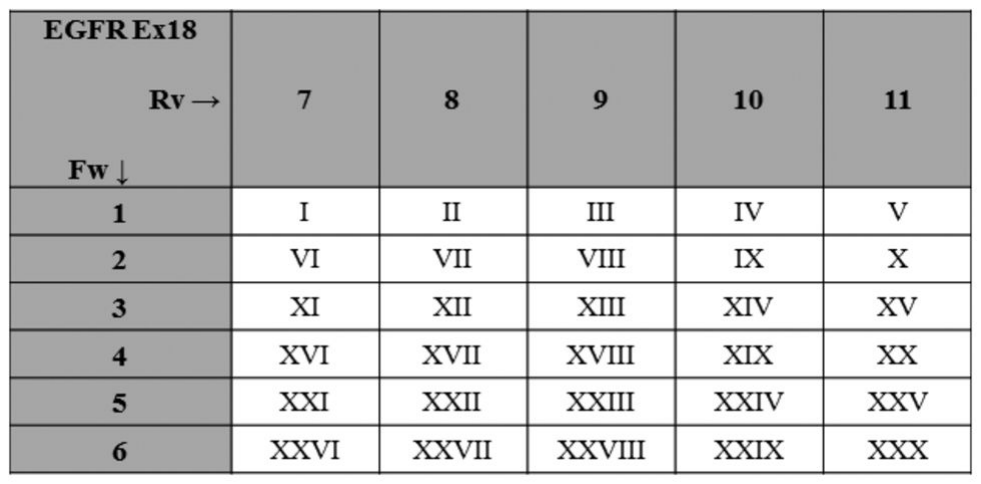
Multiple Identifier (MID) grid scheme. To ensure that in a given 454 next generation sequencing run a specific target sequence is associated with a unique pair of MID we used grid schemes; the MID pairs for EGFR exon 18 are illustrated; roman numerals indicate individual patient samples. Ex, Exon; Fw, Forward; Rv, Reverse.

Considering that each target sequence - exon and patient specific - is univocally identified by a specific couple of MIDs, at least 5 forward primers and at least 6 reverse primers with unique MIDs were necessary to analyze 30 cases in the same run (or viceversa at least 6 forward primers and at least 5 reverse ones). We used grid schemes per each *EGFR* exon to identify the unique association between MID couples and specific target sequences (see Figure 1). Both forward and reverse strand sequences (“reads”) were evaluated after parallel amplification of the target DNA. The sequences obtained were analyzed using the Amplicon Variant Analyzer (AVA) Software (Roche Diagnostics, Mannheim, Germany). Only nucleotide variations observed in both strands were considered for mutational call. Ambiguous base calls associated to stretches of homopolymer 4 base pair or longer were not considered mutated due to the limitations of the pyrosequencing chemistry that is used by 454 NGS for sequence analysis [47,48]. 

#### Analytical sensitivity

The analytical sensitivity of our 454 sequencing workflow format for *EGFR* mutational analysis was tested by serially diluting (1:1, 1:2, 1:10, 1:100, 1:1000) DNA from a pool of samples harboring a homozygous nucleotide polymorphism G>A at the 2470 position of the *EGFR* sequence (c2470 G>A, CAG CAA, Q787Q exon 20) in a pool of samples that did not harbor the nucleotide substitution (c2470, CAG). Each analysis was repeated at least twice.

#### Minimal amount of input DNA at the analytical sensitivity threshold

The input DNA at the analytical sensitivity threshold was serially diluted in H_2_O to determine the minimal amount of DNA necessary for mutation detection.

#### 454: Next Generation Sequencing reproducibility

Inter-assay reproducibility (i.e. the consistency of results with the same protocol in different runs) was assayed by repeating the sequence analysis of 20 DNA samples that were previously characterized for their *EGFR* mutational status (11 wild type cases and 9 *EGFR* mutated ones – 7 with a deletion in exon 19, one with the L858R and one with the T790M mutations). 

## Results

### Performance of the 454 Next Generation Sequencing protocol

#### Analytical sensitivity and definition of the threshold for mutational call

The analytical sensitivity of 454 NGS depends on the total number of reads that can be obtained for a given sample. Following our 454 sequencing format protocol, that targets a putative number of approximately 700 reads per amplicon, we tested a serial (1:1, 1:2, 1:10, 1:100, 1:1000) dilution of DNA with the c.2470 G>A nucleotide substitution at the 2470 polymorphic site of the *EGFR* gene sequence in a pool of human DNA without the substitution (c.2470 G).

**Figure 2:**
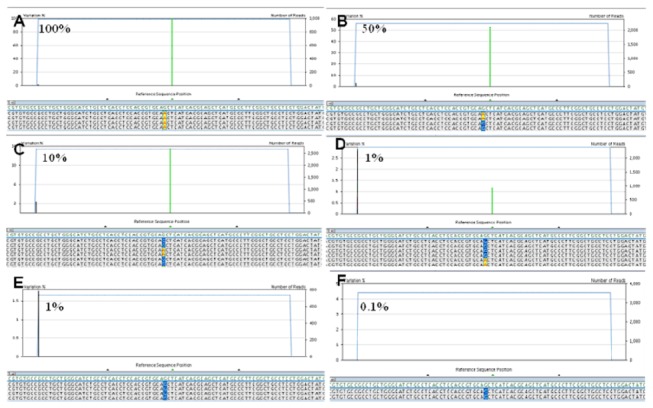
Analytical sensitivity and definition of threshold for mutational call. A-F) the polymorphic c.2470 G>A substitution was observed with the following dilutions of DNA not carrying the polymorphism: 1:1 (A), 1:2 (B), 1:10 (C), 1:100 (D), but not at 1:1000 (F). At 1:100 dilution the c.2470 G>A substitution was observed when the total number of reads allowed to detect at least 10 reads with the c.2470 G>A change (this means at least 1,000 total reads) (D). The c.2470 G>A substitution was not observed when the total number of reads was not sufficient to detect at least 10 reads with the c.2470 G>A change (E).

The c.2470 G>A substitution was consistently detected down to a 1:100 dilution only if at least 10 consensual c.2470 G>A nucleotide reads were obtained after parallel 454 sequencing. This observation is illustrated in Figure 2. With a 1:100 dilution of c.2470 G>A DNA the nucleotide substitution was observed when the total number of reads was 2,359, corresponding to 23 consensual c.2470 G>A sequences (Figure 2D). It was not observed when the total number of reads was 750, that would have corresponded to 7 consensual c.2470 G>A sequences (Figure 2E). The c.2470 G>A substitution was also not observed with a 1:1000 dilution, even when the total number of reads analyzed was very high (between 3000 and 4000), but not enough to reach 10 c.2470 G>A reads that would have required a total of 10,000 reads per amplicon (Figure 2F).

Based on these observations we established as criteria to define a sample mutated the identification of the mutation in at least 10 reads (i) *and* in at least 1% of the total number of reads analyzed (ii). The requirement of 10 consensual reads makes the criteria for the mutational call more stringent for samples that generate a total number of reads lower than expected, thus ensuring the specificity of the results.

#### Minimal amount of input DNA at the analytical sensitivity threshold

The amount DNA required to detect c.2470 G>A DNA at the analytical sensitivity threshold of 1% (1:100 c.2470 G>A DNA dilution) was serially decreased starting from 10 ng, to determine the minimal input DNA necessary to detect the nucleotide substitution. A minimal amount of 2 ng of DNA was sufficient to consistently obtain amplifiable DNA and detect the c.2470 G>A substitution. Considering that each human diploid cell contains 7 pg of DNA, 2 ng of a 1:100 dilution of c.2470 G>A DNA in c.2470_G DNA correspond to the detection of approximately 4 mutated cells in a total of 200 cells without the mutation, assuming that the mutation is heterozygous and *EGFR* dysomic.

#### Reproducibility

To evaluate the reproducibility of 454 parallel sequencing we utilized 20 DNA samples previously characterized for their *EGFR* mutational status: 11 were *EGFR* wild type, 7 had an exon 19 deletion, and one each had a L858R-exon 21 mutation and a T790M-exon 20 mutation. Each sample was repeated twice with 454 NGS and in all cases the mutational status was confirmed. In samples that harbored *EGFR* mutations the percentage of mutated reads varied on average 2.6% (median variation 1.45%, range 0. 6%- 8.6%).

### Pathologic diagnosis and microscopic evaluation of tumor cellularity in non-small cell lung carcinoma samples

Eighty cases were studied. Pathologic diagnoses are summarized in Table 2. Fifty-six of 80 cases were primary lung lesions diagnosed as adenocarcinoma (n= 33) or NSCLC not otherwise specified (NOS) (n= 23). Twenty-two specimens were from tumor metastases: 18 in lymph nodes, 2 in the pleura, and 2 in the bone. Two samples were cytology preparations from pleural effusion.

**Table 2:** Clinicopathological data of analyzed samples.

**Biopsy Samples**	**Diagnosis**	**Number of Neoplastic Cells^*a*^**	**Tumor cell enrichment**
N=14			
	14 Adenocarcinoma	848-42,504 (median: 11,828)	15-80% (median: 60.0%)
	*9 Primary*		
	*5Metastasis (LN 3, Bone 2)*		
**Cytology Samples**	**Diagnosis**	**Number of Neoplastic Cells^*b*^**	**Tumor cell enrichment**
N=66		190-730,000 (median: 11,200)	5-80% (median: 42.5%)
	38 Adenocarcinoma	952-228,150 (median: 3,956)	10-80% (median: 32.5%)
	*24Primary*		
	*12Metastasis (LN 10, Pleura 2)*		
	*2 Pleural Effusion*		
	28 NSCLC	190-730,000 (median: 18,000)	5-80% (median: 50.0%)
	*23Primary*		
	*5 Metastasis (LN 5)*		
**Total Samples**	**Diagnosis**	**Number of Neoplastic Cells*^a^*,*^b^***	**Tumor cell enrichment**
N=80		190-730,000 (median: 17,400)	5-80% (median: 50.0%)
	52 Adenocarcinoma	848-228,150 (median: 5,000)	10-80% (median: 45.0%)
	*33 Primary*		
	*17Metastasis (LN 13, Pleura 2, Bone 2)*		
	*2 Pleural Effusion*		
	28 NSCLC	190-730,000 (median: 18,000)	5-80% (median: 50.0%)
	*23Primary*		
	*5 Metastasis (LN 5)*		

***^a^***Number of neoplastic cells was evaluated in all 14 biopsy samples; **^*b*^**Number of neoplastic cells was evaluated in 39 of 66 cytology samples; quantitative estimation of the proportion of neoplastic cells was performed on all 80 cases. LN, Lymph Node; NSCLC, Non Small Cell Lung Carcinoma.

**Figure 3:**
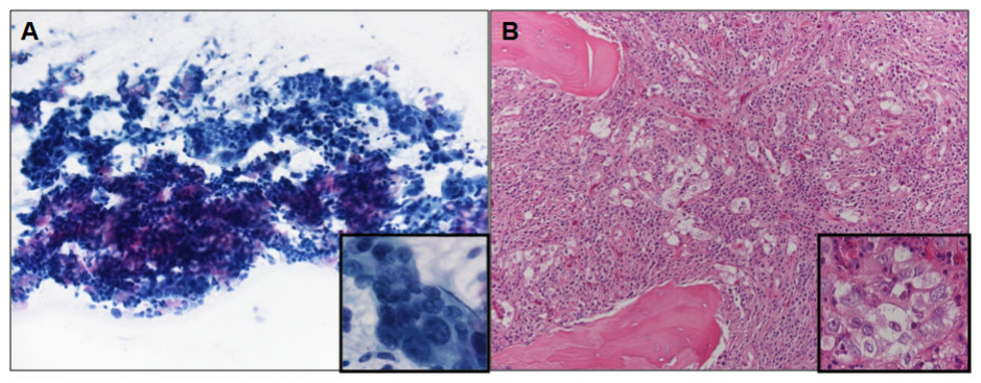
Microscopic evaluation of tumor cellularity in non-small cell lung carcinoma samples. A) cytology specimen from a 72 year old woman with adenocarcinoma metastatic to a mediastinal lymph node (May Grumwald Giemsa, 200X magnification, inset 600X); the proportion of neoplastic cells in the sample is 35%; DNA analysis was wild type after Sanger sequencing, but NGS showed two *EGFR* mutations (G721W, R831H) (case 57 of Table 5). B) biopsy specimen from a 65 year old man with adenocarcinoma metastatic to bone (vertebral body) (Hematoxylin and Eosin, 200X magnification, inset 600X); the proportion of neoplastic cells in the sample is 5%; DNA analysis was wild type after Sanger sequencing, but NGS showed the L858R *EGFR* mutation (case 80 of Table 5).

The results of the evaluation of tumor cellularity are summarized in Table 2 and illustrated in Figure 3. The proportion of neoplastic cells/total number of cells (i.e. tumor cell enrichment) was evaluated on all cases. Percentages of neoplastic cells ranged from 5 to 80% (mean 45.2%, median 50.0%). In 35 samples the proportion of neoplastic cells was less than 40%. In half of the cases the proportion of neoplastic cells was less than 50%. The total number of neoplastic cells in the sample submitted to *EGFR* mutational analysis was estimated in 53 cases. It ranged between 190 and 730,000 (mean 68,147, median 17,400). A numerical estimate of tumor cell enrichment and of the total number of neoplastic cells analyzed for *EGFR* mutation was available in all but two cases with discrepant results between Sanger and next generation sequencing (see below).

### EGFR mutational status

#### Sanger sequencing

**Figure 4:**
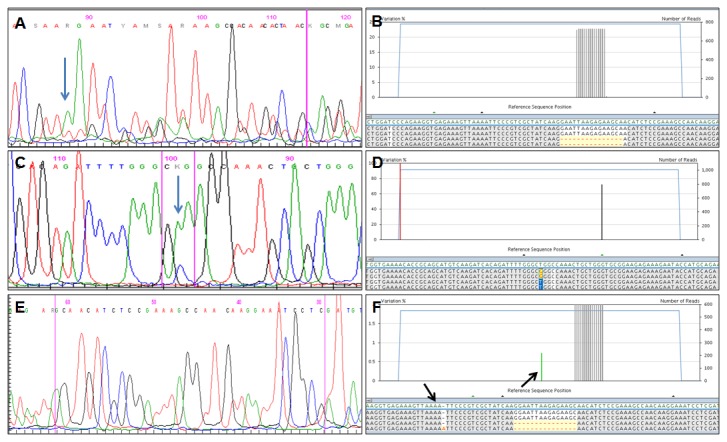
Results of sequencing analysis of *EGFR* gene after Sanger (A-C-E) and Next Generation (B-D-F) sequencing. A-B) an exon 19 deletion detected by both Sanger (A) and NGS (B); the percentage of mutated alleles identified by NGS was >20%. C-D) the L858R detected by both Sanger (C) and NGS (D); the percentage of mutated alleles identified by NGS was >20%. E-F) an exon 19 deletion detected by NGS (F) but not by the Sanger method (E); the percentage of mutated alleles identified by NGS was <20%. Blue arrows in A and in C indicate the starting nucleotide of deletion and the mutated nucleotide, respectively; black arrows in F indicate homopolymer stretches (e.g. four adenines).

**Figure 5:**
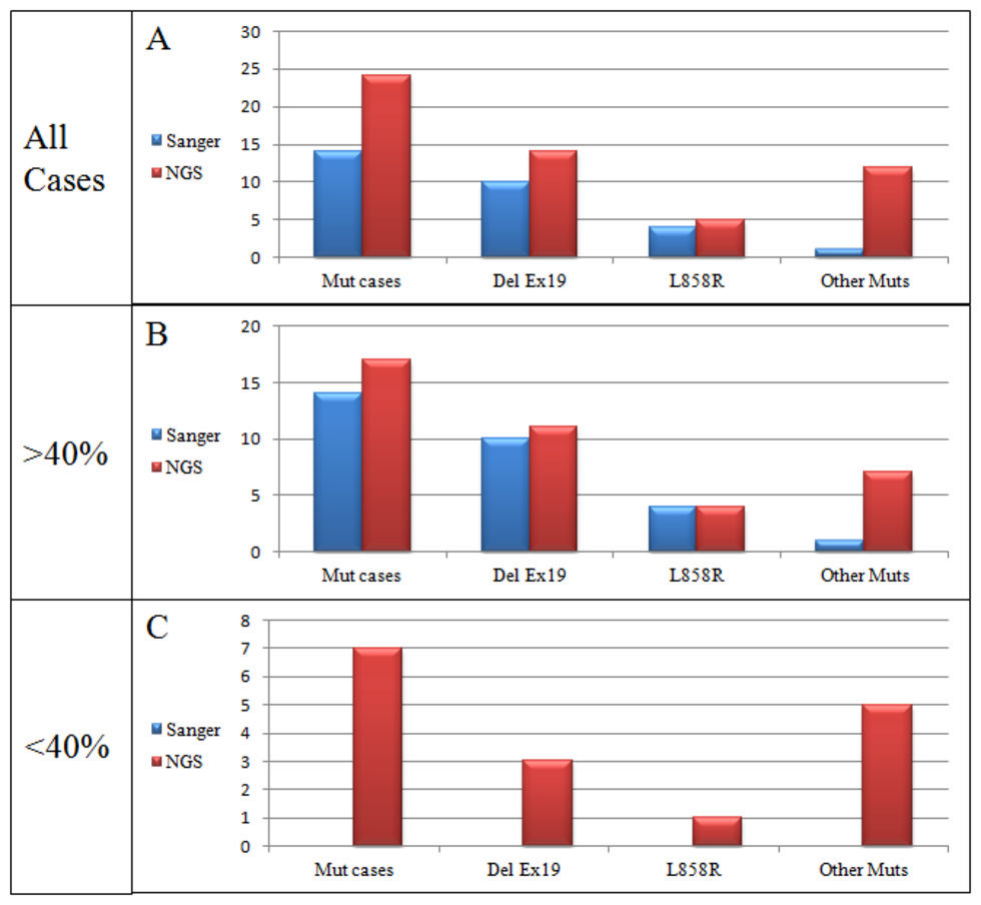
Schematic illustrating mutations detected by Sanger and Next Generation sequencing versus the proportion of neoplastic cells in the sample. NGS, Next Generation Sequencing; Mut cases, cases with any mutation; Del Ex19, deletion in exon 19; Other muts, mutations other than exon 19 deletion or L858R.

The results of Sanger sequencing are summarized in Tables 3, >4 and 5 and illustrated in Figures 4 and 5. Mutations were identified in 14 of 80 cases (17.5%) using Sanger sequencing. A total of 15 mutations were identified: 10 were exon 19 deletions, 3 were L858R (exon 21), one was G719A (exon 18) and one was a T790M mutation (exon 20). In one case there was a double T790M and L858R mutation. Mutations were found in 9 of 52 (17.3%) cases diagnosed as adenocarcinoma and in 5 of 28 (17.8%) cytology samples that could not be further subtyped and were diagnosed as NSCLC. They were found in 3 of 14 (21.4%) biopsy specimens and in 11 of 66 (16.7%) cytology samples Sanger sequencing detected mutations over a wide range of neoplastic cell number. One exon 19 deletion (del L747-A752) was identified in the specimen with the lowest number of neoplastic cells in our series (190 neoplastic cells). However, in all cases where Sanger sequencing detected *EGFR* mutations the proportion of neoplastic cells in the sample was > 40% (Table 3, Figures 4 and 5).

**Table 3:** EGFR mutational analysis using Sanger and Next Generation sequencing.

**Biopsy Samples**	**Number of mutated cases**		**Total Mutations observed**		**Mutations observed in samples with >40% of neoplastic cells**		**Mutations observed in samples with <40% of neoplastic cells**	
	*Sanger (%)*	*NGS (%)*	*Sanger*	*NGS*	*Sanger*	*NGS*	*Sanger*	*NGS*
N=14	3 (21.4)**^*a*^**	6 (42.9)**^*b*^**	4	9	4**^*a*^**	8**^*b*^**	0	1
*Del Ex19*	*2*	*2*	*2*	*2*	*2*	*2*	*0*	*0*
*L858R*	*1*	*2*	*1*	*2*	*1*	*1*	*0*	*1*
*Other muts.*	*1*	*5*	*1*	*5*	*1*	*5*	*0*	*0*
**Cytology Samples**	**Number of mutated cases**		**Total Mutations observed**		**Mutations observed in samples with >40% of neoplastic cells**		**Mutations observed in samples with <40% of neoplastic cells**	
	*Sanger (%)*	*NGS (%)*	*Sanger*	*NGS*	*Sanger*	*NGS*	*Sanger*	*NGS*
N=66	11 (16.7)	18 (27.3)**^*c*^**	11	22	11	14**^*c*^**	0	8**^*d*^**
*Del Ex19*	*8*	*12*	*8*	*12*	*8*	*9*	*0*	*3*
*L858R*	*3*	*3*	*3*	*3*	*3*	*3*	*0*	*0*
*Others muts.*	*0*	*7*	*0*	*7*	*0*	*2*	*0*	*5*
**All Samples**	**Number of mutated cases**		**Total Mutations observed**		**Mutations observed in samples with >40% of neoplastic cells**		**Mutations observed in samples with <40% of neoplastic cells**	
	*Sanger (%)*	*NGS (%)*	*Sanger*	*NGS*	*Sanger*	*NGS*	*Sanger*	*NGS*
**N=80**	**14 (17.5)^*a*^**	**24 (30.0)^*d*^**	**15**	**31**	**15^*a*^**	**22^*c*^**	**0**	**9^*b*^**

***^a^***In one case double mutations were observed; **^*b*^**in two cases double/multiple mutations were observed; **^*c*^**in four cases double/multiple mutations were observed; **^*d*^**in six cases double/multiple mutations were observed. NGS, Next Generation Sequencing; Del Ex19, deletion in exon 19; Other muts, mutations other than exon 19 deletion or L858R.

**Table 4:** Type of EGFR mutations observed in our series.

		**SANGER**		
**Type of mutation**	**Ex**	**Num cases ^*a*^**	**Predicted role for TKI treatment**	**References**
G719A	18	1	Response	[1,2,15,17]
Exon 19 deletions	19	10	Response	[1,2,8,13,15-17]
L858R	21	3	Response	[1,14-16]
T790M	20	1	Resistance	[19-21]
		**NGS**		
**Type of mutation**	**Ex**	**Num cases ^*b*^**	**Predicted role for TKI treatment**	**References**
G719A	18	1	Response	[1,2,15,17]
Exon 19 deletions	19	14	Response	[1,2,8,13,15-17]
L858R	21	5	Response	[1,14-16]
P772S	20	1	Response (Putative)	[18]
T790M	20	1	Resistance	[19-21]
R831H	21	1	Resistance	[22]
F795S	20	1	Undefined (reported in CRC and SCC of the oral cavity)	[62,65]
T785I	20	1	Undefined (reported in NSCLC)	[63,66]
V845M	21	1	Undefined (reported in adrenocortical carcinoma)	[64]
P691T, K708N, G721W, S752F, D807G	18, 19, 20, 21	1 each	Mutations not previously described	-

***^a^***In one case double mutations were observed; **^*b*^**in six cases double/multiple mutations were observed. TKI, tyrosine kinase inhibitor; CRC, colorectal carcinoma; SCC, squamous cell carcinoma; NSCLC non small cell lung carcinoma.

**Table 5:** Cases with mutations detected by Next Generation Sequencing but not by Sanger sequencing.

**# Case**	**Total tumor cell number**	**Tumor cell %**	***EGFR* status (Ex18-21) according to Sanger sequencing**	***EGFR* status (Ex18-21) according to NGS**	**Ex**	**% of mutated reads**	**Predicted role for TKI treatment**	**Clinical relevance of mutations detected by NGS but not by Sanger**	**Specimen Type**	**Diagnosis**
67	1,392	5	WT	del E746-A751	19	2	RESPONSIVE	YES	C	NSCLC
80	858	5	WT	L858R	21	6	RESPONSIVE	YES	B	ACA Bone Met
63	3,240	10	WT	del K745-A750	19	2	RESPONSIVE	YES	C	ACA
76	952	15	WT	del E746-A750	19	2	RESPONSIVE	YES	C	ACA
39	3,720	25	WT	D807G	20	16	Mut. not described	NO	C	NSCLC
57	4,672	35	WT	G721W	20	4	Mut. not described		C	ACA LN Met
				R831H	21	3	RESISTANCE	YES		
25	NA	<40	WT	S752F	20	5.2	Mut. not described	NO	C	ACA
				T785I	21	10	UNDEFINED			
30	NA	>40		F795S	20	16	UNDEFINED	NO	C	NSCLC
			L858R	L858R	21	20	RESPONSIVE			
2	228,150	46	del E746-A750	del E746-A750	19	49	RESPONSIVE	NO	C	ACA
				P772S	20	1	RESPONSIVE			
68	1,390	50		P691T	18	2.4	Mut. not described		B	ACA
			T790M	T790M	20	26	RESISTANCE	NO		
			L858R	L858R	21	26	RESPONSIVE			
79	18,000	50	WT	del L747-A750 insP	19	4	RESPONSIVE	YES	C	NSCLC LN Met
62	42,504	60		K708N	18	2.5	Mut. not described	NO	B	ACA LN Met
			G719A	G719A	18	38	RESPONSIVE			
59	20,784	80	WT	V845M	21	1.5	UNDEFINED	NO	B	ACA Trachea Met
66	11,410	80	WT	del L747-S752	19	1.7	RESPONSIVE	YES	B	ACA

Ex, Exon; B, biopsy sample; C, cytology sample; LN, lymph node; Met, metastasis; NSLC, non-small cell Lung carcinoma; ACA, adenocarcinoma; NA, not available; WT, wild type; del, deletion; ins, insertion; mut., mutation.

#### 454: Next Generation Sequencing

Each target sequence was analyzed at least 100 times per sample, ranging from 101 to 2,656 reads per target (mean 521.8). The criteria outlined above of at least 10 reads with a consensual mutation in at least 1% the total number of reads were used to diagnose all mutations. Nucleotide variations associated to homopolymer stretches were observed, usually in only one strand (Figure 4F). They were considered technical artifacts resulting from the pyrosequencing chemistry used by 454 NGS for sequence analysis, that does not adequately discriminate repeated sequences of the same nucleotide.

The results of 454 NGS are summarized in Tables 3, 4 and 5, and illustrated in Figures 4 and 5. Raw data are available in the Sequence Read Archive (SRA) (http://www.ncbi.nlm.nih.gov/sra/), accession number SRP030636. 

Mutations were identified in 24 of 80 cases (30.0%) using 454 NGS. A total of 31 mutations were identified, and all those identified using Sanger sequencing were confirmed. In addition, 454 NGS detected 4 exon 19 deletions, 2 L858R (exon 21), and a variety of other individual mutations (P691T, K708N, G721W, S752F, P772S, T785I, F795S, D807G, R831H, V845M). These mutations were searched in the online databases COSMIC (http://www.sanger.ac.uk/genetics/CGP/cosmic/) and Somatic Mutations in Epidermal Growth Factor Receptor Database (SM-EGFR-DB) (http://www.somaticmutations-egfr.info/). Five mutations (P691T, K708N, G721W, S752F, D807G) have not been previously described.

C-T or G-A transitions have been associated with sequencing artifacts in FFPE samples with low amounts of DNA [49]. Of our five mutations with no previous record in literature databases one (S752F) was a C → T transition (TCT → TTT), no mutation was a G → A transition.

The relevance for patient treatment with tyrosine kinase inhibitors of all mutations found in the study is summarized in Table 4.

Mutations were found in 16 of 52 (30.8%) cases diagnosed as adenocarcinoma and in 8 of 28 (28.6%) cytology samples that could not be further subtyped and were diagnosed as NSCLC. They were found in 6 of 14 (42.9%) biopsy specimens and in 18 of 66 (27.3%) cytology samples.

454 NGS detected mutations over a wide range of neoplastic cell number. The exon 19 deletion (del L747-A752) identified by Sanger sequencing in the specimen with the lowest number of neoplastic cells in our series (190 neoplastic cells) was also identified by 454 NGS. 454 NGS detected 22 *EGFR* mutations in cases with a proportion of neoplastic cells in the sample analyzed > 40% and 9 mutations in cases with a low proportion of neoplastic cells (

< 40%) (Table 3, Figures 4 and 5).

In six cases two *EGFR* mutations (five cases) or three *EGFR* mutations (one case) were observed in the same specimen. In one case (Table 5, case 62) two mutations of the same exon (exon 18) were identified in different DNA strands (not in haploptype). In the remaining 5 cases mutations where located in different exons. In one of these 5 cases the percentage of mutated reads was identical for both mutations (T790M and L858R) (Table 5, case 68), and therefore compatible with the same population of neoplastic cells harboring both nucleotide changes. In 2 of the 5 cases the percentage of mutated reads was very similar (Table 5, case 57 and 30), suggesting that it is the same population of neoplastic cells to harbor more than one mutation.

**Figure 6:**
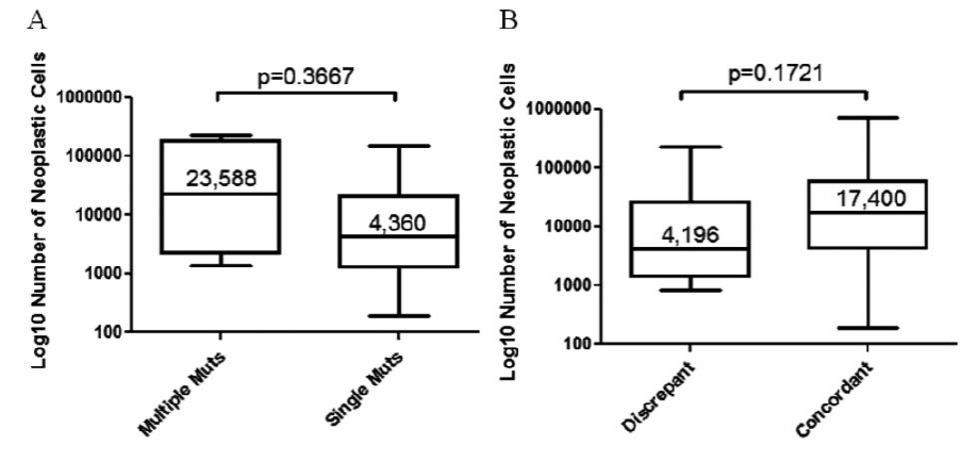
Tumor cell number in cases with multiple vs. **single *EGFR* mutation (A) and in cases where NGS identified mutations not seen after Sanger sequencing (discrepant cases) (B)**. Numbers refer to the median number of neoplastic cells in the sample; bars indicate the smallest and highest number of neoplastic cells; the p-value was obtained with the student T test.

The median number of neoplastic cells was 23,588 (range 1,390 - 228,150) in the cases with multiple mutations and was 4,360 (range 190 - 154,000) in those where a single mutation was detected. The difference in the number of neoplastic cells between the two groups was not statistically significant (p=0.3667) (Figure 6A).

#### Cases with differences in the EGFR mutational status following Sanger and 454 Next Generation sequencing

Among the 80 cases analyzed in this study sequencing results with Sanger and 454 NGS were identical in 66 cases, 56 cases were wild type by both methods, in 10 cases the same mutation was identified with both. Differences in the *EGFR* mutation pattern after Sanger and 454 NGS were observed in fourteen cases (17.5%) and are summarized in Table 5. One example is illustrated in Figure 4 E-F. Sanger sequencing did not detect *EGFR* mutation in 10 of these 14 cases. In these 10 cases one or more mutations were identified with 454 NGS. They included exon 19 deletions in 5 of the ten cases, as well as L858R and R831H in one case each. In the remaining 4 cases Sanger sequencing identified *EGFR* mutations (Table 5, Cases 2, 30, 62, 68). All of them (del E746-A750, L585R, G719A, T790M) were confirmed by 454 NGS that also detected additional mutations. 

The mutations detected by 454 NGS in the 14 cases were single in 8 and multiple in the other 6 cases, ranging from 2 to 3 mutations per case. In one case (Table 5, Case 68) Sanger sequencing identified two distinct mutations (T790M and L858R).

Among the 14 cases with different *EGFR* mutational status two sets could be easily recognized. In one consisting of 7 cases (Table 5, Cases 25, 39, 57, 63, 67, 76, 80) the proportion of neoplastic cells in the sample analyzed was below 40%. All these cases were considered wild type after Sanger sequencing, but 454 NGS detected *EGFR* mutations with a percentage of mutated reads that was below the analytical sensitivity threshold of Sanger sequencing that is generally set at 20% mutated alleles (40% neoplastic cells, assuming that the mutation is heterozygous and *EGFR* dysomic) (Figure 4 E-F). In the second set, also consisting of seven cases (Table 5, Cases 2, 30, 59, 62, 66, 68, 79) the proportion of neoplastic cells was greater than 40%. All four cases where Sanger sequencing detected *EGFR* mutations belong to this set. In all cases the mutations confirmed by 454 NGS had a high proportion of mutated reads (

> 20%) (Figure 4 A-D). The additional mutations detected in these cases by 454 NGS - but not by Sanger - had a low percentage of mutated reads, below the Sanger detection threshold of 20% mutated alleles: it was <5% in 6 of the 7 cases, and 16% in the remaining one

. 

In the 14 cases with different *EGFR* mutation pattern the median number of neoplastic cells in the samples analyzed was 4,196 (range 848 - 228,150). It was 17,400 (range 190 - 730,000) in the 66 cases where sequencing with the Sanger method and 454 NGS gave identical results. The difference between the two groups was not statistically significant (p=0.1721) (Figure 6B).

## Discussion

The remarkable association between certain *EGFR* mutations, especially exon 19 deletions or L858R mutation in exon 21 and clinical benefit in patients with NSCLC treated with EGFR tyrosine kinase inhibitors (TKIs), such as Gefitinib and Erlotinib, is well established [1-9,13-16,23]. Therefore the selection of lung cancer patients for molecular therapy with TKIs mandates the analysis of DNA extracted from tumor samples. Unfortunately it is difficult to achieve full control and standardization of all the pre-analytical steps related to sample collection that ultimately define the quality of the specimens that are submitted for molecular analysis. Guidelines identify a careful evaluation of tumor cell content before DNA analysis as a critical issue [10,11]. In patients with advanced lung carcinoma small biopsies or cytology specimens are frequently the only material available to establish the pathologic diagnosis and for molecular testing. In these samples the percentage of neoplastic cells is often low and to enrich the tumor cell content by dissecting tumor cells is cumbersome and often impossible. Since chemotherapy is the only available treatment for patients with advanced lung carcinoma, these samples are very crucial. Their use needs to be carefully optimized for both morphologic diagnosis and DNA analysis in spite of their suboptimal nature [26].

Therefore, while the search for methods and protocols that identify mutations with high sensitivity is one of the goals of molecular diagnosis, the issue is particularly relevant for the management of patients with lung carcinoma. Many strategies that utilize a variety of technical approaches have been developed in the recent past and are being used for diagnosis [30-39]. The application of NGS to the routine analysis of patient samples is being evaluated and may radically change the approach to the molecular diagnostics of solid tumors [50-52]. Reports on the use of NGS for the analysis of lung carcinoma are still relatively few [53-57], but show encouraging results. Although most samples analyzed in these reports have been lung resections specimens, some with limited amounts of tumor have also been studied. Remarkably, Buttitta et al. have shown that in principle NGS can identify *EGFR* mutated alleles in bronchoalveolar lavages and in the pleural fluid of samples where tumor cells were very scarce or even altogether absent after microscopic evaluation [55].

To the best of our knowledge this is the first study to systematically assess the application of NGS to the analysis of the small biopsy and cytology lung carcinoma samples that represent the majority of the specimens routinely submitted for both tumor typing and molecular analysis. All specimens were unselected and representative of the material ordinarily submitted to three referral Italian centres. The proportion of neoplastic cells - i.e. tumor cell enrichment - was evaluated on all cases and in the majority of them the absolute number of tumor cells was also be assessed.

For NGS analysis we have used the 454 GS-Junior sequencer and have defined a protocol format that targets *EGFR* exons 18, 19, 20, 21, all of which need to be evaluated according to current guidelines [10]. Our protocol is designed to analyze 100-150 amplicons per run, corresponding to 25-35 samples screened for the four *EGFR* exons and reaches an analytical sensitivity of 1%. This analytical sensitivity is equivalent to that of the most sensitive real time based PCR methods currently available that are however mutation specific and therefore not able to identify all possible *EGFR* mutations present in the sample. With the analysis of 100-150 amplicons per run and given the features of the 454 GS-Junior sequencer the theoretical re-sequencing depth is approximately of 700 reads per amplicon. We obtained an average number of ~522 reads per amplicon with adequate coverage of all exons on all samples analyzed. Should an even higher analytical sensitivity and greater re-sequencing depth be necessary in individual cases, this can easily be accomplished by decreasing the number of amplicons (i.e. patient samples) analyzed per run.

The importance of using methods that are highly sensitive is underlined by the observation that the average proportion of tumor cells in the specimens was ~45% and therefore inferior to the general threshold of 50% required to reliably diagnose mutations with Sanger sequencing. In case of a negative mutational result all these cases would have required re-testing with one of the high sensitivity methods currently available or re-biopsy to obtain a higher proportion of neoplastic cells, causing additional costs and treatment delays. This issue is clearly illustrated by the samples shown in Figure 3, representative of many of our randomly selected cases for which *EGFR* analysis was requested. In these samples a large excess of non-neoplastic elements is intermixed so closely with the neoplastic cells that tumor enrichment is impossible by manual dissection and very difficult even using laser assisted microdissection. It has to be underlined that there is no guarantee that re-biopsying the patient would give a better specimen for molecular analysis, since an excess of non-neoplastic tissue is very common in those obtained from metastatic tumor sites and in many aggressive high stage carcinomas. Interestingly, while the proportion of tumor cells in the samples submitted for molecular diagnosis was clearly an issue, the number of neoplastic cells was not. Several thousand tumor cells were present in most specimens, including cytology samples. This observation supports the findings of several studies that have shown how cytology specimens can be utilized to predict the response of patients with lung carcinoma to TKIs using a variety of molecular methods to identify mutations [30,44,58-61].

All mutations identified by conventional Sanger sequencing were also identified by targeted NGS, but the proportion of cases - 14 out of 80 - in which NGS identified at least one additional *EGFR* mutation compared with the Sanger method is considerable. Overall 16 more mutations were detected, and all were observed with a percentage of reads that was below the sensitivity of conventional sequencing. We found a very good correlation between the proportion of tumor cell content in the specimen and the results of mutational analysis, similar to a pilot study that has recently addressed this issue using 454 NGS [54]. In half of the cases where NGS identified *EGFR* mutations not seen after Sanger sequencing the proportion of neoplastic cells was below 40%. In the other half of them the proportion of neoplastic cells was adequate, but the far superior sensitivity of NGS detected mutational events not seen by the Sanger method. The issue of the absolute number of neoplastic cells in the specimens subjected to NGS analysis has not been addressed by other studies. We observed that the absolute number of neoplastic cells was lower in discrepant cases were NGS identified mutations not seen by conventional sequencing. The difference did not reach statistical significance, but it appears that the separate parallel analysis of individual nucleotide sequences allows better resolution in those specimens where the absolute number of neoplastic cells is relatively limited.

Very importantly, in six of the 14 discrepant cases mentioned above targeted NGS identified exon 19 deletions or the L858R mutation not seen after Sanger sequencing, allowing the patient to be treated with TKIs. In one additional case (Table 5, case 57) the R831H mutation associated with resistance to TKIs [22] was identified in a tumor that was *EGFR* wild type after the Sanger method. This mutation was present in a small subpopulation of cells, corresponding to 3% of mutated reads and its identification would have been very difficult with conventional sequencing methods, including pyrosequencing.

By generating a quantitative assessment of the number of mutated reads and by defining the haplotype of the nucleotide changes, targeted NGS allows to discriminate between large populations of mutated cells and small subclones and can provide useful information as to whether mutations are present in the same population of cells or not. It may thus contribute important insights into to the clonal evolution of *EGFR* mutated cases [56]. Sanger sequencing identified one case (Table 5, case 68) with two distinct mutations - the L858R predictive of response and the T790M associated with acquired resistance to TKIs treatment. Targeted NGS found both mutations in a conspicuous number of reads, confirming the result of conventional sequencing. Since both mutations had the same proportion of mutated reads they were most likely present in the same neoplastic cell population. A third, much smaller population of mutated alleles, compatible with a small neoplastic cells subclone was also identified in the same sample. In five more specimens multiple mutations were identified only by NGS. In two of them there were two nucleotide variants with a similar proportion of mutated reads in the same specimen, suggesting that both variants may have arisen in the same neoplastic cells (Table 5, case 57 and 30). In three of the five cases the relative proportions of variant alleles was more consistent with the existence of a dominant mutated neoplastic cell clone and of a small neoplastic cell subset carrying the additional mutation. Interestingly in one these cases a P772S exon 20 mutation known to be associated with TKIs treatment response was identified in a very small proportion of reads in a case with a dominant population of neoplastic cells carrying one of the exon 19 deletions typically associated with sensitivity to TKI treatment, indicating that more than one favorable mutational event may be present in the same tumor (Table 5, case 2).

Although multiple mutations were more commonly observed in cases with a large amount of neoplastic cells, the number of neoplastic cells present in the specimen – in absolute term or relative to that of the “contaminant” non-neoplastic cell population – did not correlate with the ability of NGS to identify multiple mutations.

Since targeted NGS allows to screen the entire *EGFR* exon and because of its high analytical sensitivity we have identified in our series three uncommon mutations (T785I, F795S, V845M) that have been previously reported in lung carcinoma or in other tumors types, the clinical relevance of which is currently undefined [62-66]. We have also identified five mutations that have not been previously reported (P691T, K708N, C721W, S752F, D807G). Low amounts of template DNA and formalin fixation can cause random polymerase errors in nucleotide incorporation and sequencing artifacts that are usually C-T or G-A transitions [49,67]. In the five cases with previously unreported mutations thousands of neoplastic cells were present. Only two of the five cases were formalin-fixed biopsies and in only one case – a cytology specimen fixed in alcohol – the mutation was a C → T transition. Considering that most of the knowledge collected on the spectrum of *EGFR* mutations has been acquired through conventional sequencing methods it is not surprising that novel nucleotide variations may be disclosed by highly sensitive next generation methods. One recent study using the next generation Illumina HISeq2000 sequencing platform for the analysis of routinely processed lung carcinoma samples has identified both uncommon and previously unreported mutations with a rate very similar to ours [53]. Although the meaning of these findings clearly requires additional investigation, we do not believe that unexpected sequence variants should be discounted, also considering that some of these changes can modify the response to TKIs treatment and may be markers of resistance to targeted molecular therapy [53].

Methods to test *EGFR* or other mutations in specimens with unfavorable tumor cell content need to be very sensitive [30]. Testing algorithms with parallel duplicate analysis using both conventional sequencing and a highly sensitive method have been suggested as a strategy for these specimens, but this adds to cost and turnaround time, and requires additional DNA for the analysis. Mutational analysis of *EGFR* by NGS overcomes the issue of limited tumor amounts because it is highly sensitive. If the percentage of tumor cells is established in the sample that is going to be sequenced by pre-analytical microscopic examination, quantitative NGS data allow to easily distinguish dominant mutations from alterations found only in subclonal fractions of the tumor. We have observed only small discrepancies in the proportion of mutated alleles after the repetition of samples with DNA mutations, indicating that the reproducibility of our targeted NGS protocol is more than adequate. 

It has to be pointed out that our NGS protocol requires a minimal amount of 2 ng to consistently obtain amplifiable DNA for the detection of the c.2470 G>A polymorphism and that it is effective with the quantity and quality of DNA that is currently obtained by limited formalin-fixed biopsies and routinely processed cytology samples obtained from the different medical centres that participated to this multicentric study. The only technical drawback of 454 NGS that we encountered is its inability to discriminate homopolymer sequences. This is a consequence of the pyrosequencing chemistry utilized by the 454 platform and may result in ambiguous base calls that can be misinterpreted as frame-shift mutations [47,48] (Figure 4F). Several studies are indeed demonstrating that the performance of NGS in the analysis of *routine* samples is superior to that of other sensitive techniques including conventional pyrosequencing, and highly sensitive mutation-specific methods like Therascreen and chip array hybridization [53,57,68].

One issue that may limit the application of NGS to the routine practice of molecular diagnosis is its procedure that is relatively labor intensive, and therefore unpractical for the ad hoc analysis of individual specimens as soon as they arrive to the laboratory. However, many samples can be analyzed at the same time, even for a considerable number of different genes. Our protocol - optimized for the analysis of 100-150 amplicons in one run - has been designed for the needs of a referral molecular diagnostic laboratory where requests for mutational evaluation of *EGFR* and other genes easily accumulate in a short time. Since the entire NGS analysis is accomplished in 2 working days, turnaround time requirements of 10 working days [10,11] can be effectively satisfied by grouping the evaluation of specimens in batches. Overall reagent costs per run are approximately 2,000 Euro. If 100 amplicons are analyzed in a given run the reagent cost per amplicon is 20 Euro, and even if that of technical operators is added the overall figures per sample are inferior to that of most commercially available kits for *EGFR* mutation detection.

 In conclusion, we have defined a NGS protocol based on the 454 GS-Junior platform for the analysis of *EGFR* and validated it with unselected limited tumor samples routinely submitted for molecular diagnosis to three different Italian laboratories. Targeted NGS is robust, cost-effective and greatly improves the detection of *EGFR* mutations in lung carcinoma patients. Its use should be promoted for the clinical diagnosis of mutations in specimens with unfavorable tumor cell content.

## References

[1] Lynch TJ, Bell DW, Sordella R, Gurubhagavatula S, Okimoto RA et al. (2004) Activating mutations in the epidermal growth factor receptor underlying responsiveness of non-small-cell lung cancer to gefitinib. N Engl J Med 350: 2129-2139. doi:10.1056/NEJMoa040938. PubMed: 15118073. 15118073

[2] Paez JG, Jänne PA, Lee JC, Tracy S, Greulich H et al. (2004) EGFR mutations in lung cancer: correlation with clinical response to gefitinib therapy. Science 304: 1497-1500. doi:10.1126/science.1099314. PubMed: 15118125. 15118125

[3] Fukuoka M, Wu YL, Thongprasert S, Sunpaweravong P, Leong SS et al. (2011) Biomarker analyses and final overall survival results from a phase III, randomized, open-label, first-line study of gefitinib versus carboplatin/paclitaxel in clinically selected patients with advanced non-small-cell lung cancer in Asia (IPASS). J Clin Oncol 29: 2866-2874. doi:10.1200/JCO.2010.33.4235. PubMed: 21670455. 21670455

[4] Mitsudomi T, Morita S, Yatabe Y, Negoro S, Okamoto I et al. (2010) Gefitinib versus cisplatin plus docetaxel in patients with non-small-cell lung cancer harbouring mutations of the epidermal growth factor receptor (WJTOG3405): an open label, randomised phase 3 trial. Lancet Oncol 11: 121-128. doi:10.1016/S1470-2045(09)70364-X. PubMed: 20022809. 20022809

[5] Mok TS, Wu YL, Thongprasert S, Yang CH, Chu DT et al. (2009) Gefitinib or carboplatin-paclitaxel in pulmonary adenocarcinoma. N Engl J Med 361: 947-957. doi:10.1056/NEJMoa0810699. PubMed: 19692680. 19692680

[6] Rosell R, Carcereny E, Gervais R, Vergnenegre A, Massuti B et al. (2012) Erlotinib versus standard chemotherapy as first-line treatment for European patients with advanced EGFR mutation-positive non-small-cell lung cancer (EURTAC): a multicentre, open-label, randomised phase 3 trial. Lancet Oncol 13: 239-246. doi:10.1016/S1470-2045(12)70227-9. PubMed: 22285168. 22285168

[7] Zhou C, Wu YL, Chen G, Feng J, Liu XQ et al. (2011) Erlotinib versus chemotherapy as first-line treatment for patients with advanced EGFR mutation-positive non-small-cell lung cancer (OPTIMAL, CTONG-0802): a multicentre, open-label, randomised, phase 3 study. Lancet Oncol 12: 735-742. doi:10.1016/S1470-2045(11)70184-X. PubMed: 21783417. 21783417

[8] Maemondo M, Inoue A, Kobayashi K, Sugawara S, Oizumi S et al. (2010) Gefitinib or chemotherapy for non-small-cell lung cancer with mutated EGFR. N Engl J Med 362: 2380-2388. doi:10.1056/NEJMoa0909530. PubMed: 20573926. 20573926

[9] Yang JC, Schuler MH, Yamamoto N, O'Byrne KJ, et al. (2012) LUX-Lung 3: Afatinib versus pemetrexed/cisplatin as a first-line treatment in advanced lung cancer harbouring EGFR-activating mutations. J Clin Oncol 30S: LBA7500

[10] Lindeman NI, Cagle PT, Beasley MB, Chitale DA, Dacic S et al. (2013) Molecular Testing Guideline for Selection of Lung Cancer Patients for EGFR and ALK Tyrosine Kinase Inhibitors: Guideline from the College of American Pathologists, International Association for the Study of Lung Cancer, and Association for Molecular. Pathology - Arch Pathol Lab Med: 828-860. 23551194 10.5858/arpa.2012-0720-OA PMC4162344

[11] Pirker R, Herth FJ, Kerr KM, Filipits M, Taron M et al. (2010) Consensus for EGFR mutation testing in non-small cell lung cancer: results from a European workshop. J Thorac Oncol 5: 1706-1713. doi:10.1097/JTO.0b013e3181f1c8de. PubMed: 20871269. 20871269

[12] Marks JL, Broderick S, Zhou Q, Chitale D, Li AR et al. (2008) Prognostic and therapeutic implications of EGFR and KRAS mutations in resected lung adenocarcinoma. J Thorac Oncol 3: 111-116. doi:10.1097/JTO.0b013e318160c607. PubMed: 18303429. 18303429

[13] Eberhard DA, Johnson BE, Amler LC, Goddard AD, Heldens SL et al. (2005) Mutations in the epidermal growth factor receptor and in KRAS are predictive and prognostic indicators in patients with non-small-cell lung cancer treated with chemotherapy alone and in combination with erlotinib. J Clin Oncol 23: 5900-5909. doi:10.1200/JCO.2005.02.857. PubMed: 16043828. 16043828

[14] Han SW, Kim TY, Hwang PG, Jeong S, Kim J et al. (2005) Predictive and prognostic impact of epidermal growth factor receptor mutation in non-small-cell lung cancer patients treated with gefitinib. J Clin Oncol 23: 2493-2501. doi:10.1200/JCO.2005.01.388. PubMed: 15710947. 15710947

[15] Huang SF, Liu HP, Li LH, Ku YC, Fu YN et al. (2004) High frequency of epidermal growth factor receptor mutations with complex patterns in non-small cell lung cancers related to gefitinib responsiveness in Taiwan. Clin Cancer Res 10: 8195-8203. doi:10.1158/1078-0432.CCR-04-1245. PubMed: 15623594. 15623594

[16] Johnson BE, Jänne PA (2005) Epidermal growth factor receptor mutations in patients with non-small cell lung cancer. Cancer Res 65: 7525-7529. PubMed: 16140912. 16140912 10.1158/0008-5472.CAN-05-1257

[17] Rosell R, Ichinose Y, Taron M, Sarries C, Queralt C et al. (2005) Mutations in the tyrosine kinase domain of the EGFR gene associated with gefitinib response in non-small-cell lung cancer. Lung Cancer 50: 25-33. doi:10.1016/j.lungcan.2005.05.017. PubMed: 16011858. 16011858

[18] Sequist LV, von Pawel J, Garmey EG, Akerley WL, Brugger W et al. (2011) Randomized phase II study of erlotinib plus tivantinib versus erlotinib plus placebo in previously treated non-small-cell lung cancer. J Clin Oncol 29: 3307-3315. doi:10.1200/JCO.2010.34.0570. PubMed: 21768463. 21768463

[19] Balak MN, Gong Y, Riely GJ, Somwar R, Li AR et al. (2006) Novel D761Y and common secondary T790M mutations in epidermal growth factor receptor-mutant lung adenocarcinomas with acquired resistance to kinase inhibitors. Clin Cancer Res 12: 6494-6501. doi:10.1158/1078-0432.CCR-06-1570. PubMed: 17085664. 17085664

[20] Kosaka T, Yatabe Y, Endoh H, Yoshida K, Hida T et al. (2006) Analysis of epidermal growth factor receptor gene mutation in patients with non-small cell lung cancer and acquired resistance to gefitinib. Clin Cancer Res 12: 5764-5769. doi:10.1158/1078-0432.CCR-06-0714. PubMed: 17020982. 17020982

[21] Pao W, Miller VA, Politi KA, Riely GJ, Somwar R et al. (2005) Acquired resistance of lung adenocarcinomas to gefitinib or erlotinib is associated with a second mutation in the EGFR kinase domain. PLoS Med 2: e73. doi:10.1371/journal.pmed.0020073. PubMed: 15737014. 15737014 PMC549606

[22] Yang CH, Yu CJ, Shih JY, Chang YC, Hu FC et al. (2008) Specific EGFR mutations predict treatment outcome of stage IIIB/IV patients with chemotherapy-naive non-small-cell lung cancer receiving first-line gefitinib monotherapy. J Clin Oncol 26: 2745-2753. doi:10.1200/JCO.2007.15.6695. PubMed: 18509184. 18509184

[23] Rosell R, Moran T, Queralt C, Porta R, Cardenal F et al. (2009) Screening for epidermal growth factor receptor mutations in lung cancer. N Engl J Med 361: 958-967. doi:10.1056/NEJMoa0904554. PubMed: 19692684. 19692684

[24] Travis WD, Brambilla E, Müller-Hermelink HK, Harris CC (2004) WHO. Pathology and Genetics of Tumours of the Lung, Pleura,Thymus and Heart; Tumours W Lyon, France: IARC.

[25] Beau-Faller M, Degeorges A, Rolland E, Mounawar M, Antoine M et al. (2011) Cross-validation study for epidermal growth factor receptor and KRAS mutation detection in 74 blinded non-small cell lung carcinoma samples: a total of 5550 exons sequenced by 15 molecular French laboratories (evaluation of the EGFR mutation status for the administration of EGFR-TKIs in non-small cell lung carcinoma [ERMETIC] project--part 1). J Thorac Oncol 6: 1006-1015. doi:10.1097/JTO.0b013e318211dcee. PubMed: 21532509. 21532509

[26] Aisner DL, Marshall CB (2012) Molecular pathology of non-small cell lung cancer: a practical guide. Am J Clin Pathol 138: 332-346. doi:10.1309/AJCPFR12WJKCEEZZ. PubMed: 22912349. 22912349

[27] John T, Liu G, Tsao MS (2009) Overview of molecular testing in non-small-cell lung cancer: mutational analysis, gene copy number, protein expression and other biomarkers of EGFR for the prediction of response to tyrosine kinase inhibitors. Oncogene 28 Suppl 1: S14-S23. doi:10.1038/onc.2009.197. PubMed: 19680292. 19680292

[28] Eberhard DA, Giaccone G, Johnson BE (2008) Biomarkers of response to epidermal growth factor receptor inhibitors in Non-Small-Cell Lung Cancer Working Group: standardization for use in the clinical trial setting. J Clin Oncol 26: 983-994. doi:10.1200/JCO.2007.12.9858. PubMed: 18281673. 18281673

[29] Arcila M, Lau C, Nafa K, Ladanyi M (2011) Detection of KRAS and BRAF mutations in colorectal carcinoma roles for high-sensitivity locked nucleic acid-PCR sequencing and broad-spectrum mass spectrometry genotyping. J Mol Diagn 13: 64-73. doi:10.1016/j.jmoldx.2010.11.005. PubMed: 21227396. 21227396 PMC3070595

[30] Ellison G, Zhu G, Moulis A, Dearden S, Speake G et al. (2013) EGFR mutation testing in lung cancer: a review of available methods and their use for analysis of tumour tissue and cytology samples. J Clin Pathol 66: 79-89. doi:10.1136/jclinpath-2012-201194. PubMed: 23172555. 23172555 PMC3582044

[31] Nomoto K, Tsuta K, Takano T, Fukui T, Yokozawa K et al. (2006) Detection of EGFR mutations in archived cytologic specimens of non-small cell lung cancer using high-resolution melting analysis. Am J Clin Pathol 126: 608-615. doi:10.1309/N5PQNGW2QKMX09X7. PubMed: 16938658. 16938658

[32] Kawada I, Soejima K, Watanabe H, Nakachi I, Yasuda H et al. (2008) An alternative method for screening EGFR mutation using RFLP in non-small cell lung cancer patients. J Thorac Oncol 3: 1096-1103. doi:10.1097/JTO.0b013e318186fadd. PubMed: 18827604. 18827604

[33] van Eijk R, Licht J, Schrumpf M, Talebian Yazdi M, Ruano D et al. (2011) Rapid KRAS, EGFR, BRAF and PIK3CA mutation analysis of fine needle aspirates from non-small-cell lung cancer using allele-specific qPCR. PLOS ONE 6: e17791. doi:10.1371/journal.pone.0017791. PubMed: 21408138. 21408138 PMC3050927

[34] Miyamae Y, Shimizu K, Mitani Y, Araki T, Kawai Y et al. (2010) Mutation detection of epidermal growth factor receptor and KRAS genes using the smart amplification process version 2 from formalin-fixed, paraffin-embedded lung cancer tissue. J Mol Diagn 12: 257-264. doi:10.2353/jmoldx.2010.090105. PubMed: 20093389. 20093389 PMC2871734

[35] Araki T, Shimizu K, Nakamura T, Baba M, Kawai Y et al. (2011) Clinical screening assay for EGFR exon 19 mutations using PNA-clamp smart amplification process version 2 in lung adenocarcinoma. Oncol Rep 26: 1213-1219. PubMed: 21769434. 21769434 10.3892/or.2011.1391

[36] Dufort S, Richard MJ, Lantuejoul S, de Fraipont F (2011) Pyrosequencing, a method approved to detect the two major EGFR mutations for anti EGFR therapy in NSCLC. J Exp Clin Cancer Res 30: 57. doi:10.1186/1756-9966-30-57. PubMed: 21575212. 21575212 PMC3120717

[37] Yu J, Kane S, Wu J, Benedettini E, Li D et al. (2009) Mutation-specific antibodies for the detection of EGFR mutations in non-small-cell lung cancer. Clin Cancer Res 15: 3023-3028. doi:10.1158/1078-0432.CCR-08-2739. PubMed: 19366827. 19366827

[38] Kato Y, Peled N, Wynes MW, Yoshida K, Pardo M et al. (2010) Novel epidermal growth factor receptor mutation-specific antibodies for non-small cell lung cancer: immunohistochemistry as a possible screening method for epidermal growth factor receptor mutations. J Thorac Oncol 5: 1551-1558. doi:10.1097/JTO.0b013e3181e9da60. PubMed: 20697298. 20697298 PMC2946481

[39] Angulo B, Conde E, Suárez-Gauthier A, Plaza C, Martínez R et al. (2012) A comparison of EGFR mutation testing methods in lung carcinoma: direct sequencing, real-time PCR and immunohistochemistry. PLOS ONE 7: e43842. doi:10.1371/journal.pone.0043842. PubMed: 22952784. 22952784 PMC3428292

[40] Whitehall V, Tran K, Umapathy A, Grieu F, Hewitt C et al. (2009) A multicenter blinded study to evaluate KRAS mutation testing methodologies in the clinical setting. J Mol Diagn 11: 543-552. doi:10.2353/jmoldx.2009.090057. PubMed: 19815694. 19815694 PMC2765753

[41] Angulo B, García-García E, Martínez R, Suárez-Gauthier A, Conde E et al. (2010) A commercial real-time PCR kit provides greater sensitivity than direct sequencing to detect KRAS mutations: a morphology-based approach in colorectal carcinoma. J Mol Diagn 12: 292-299. doi:10.2353/jmoldx.2010.090139. PubMed: 20203003. 20203003 PMC2860464

[42] Metzker ML (2010) Sequencing technologies - the next generation. Nat Rev Genet 11: 31-46. doi:10.1038/nrg2626. PubMed: 19997069. 19997069

[43] Meyerson M, Gabriel S, Getz G (2010) Advances in understanding cancer genomes through second-generation sequencing. Nat Rev Genet 11: 685-696. doi:10.1038/nrg2841. PubMed: 20847746. 20847746

[44] Malapelle U, Bellevicine C, De Luca C, Salatiello M, De Stefano A et al. (2013) EGFR mutations detected on cytology samples by a centralized laboratory reliably predict response to gefitinib in non-small cell lung carcinoma patients. Cancer Cytopathol, 121: 552–60. PubMed: 23780873. 23780873 10.1002/cncy.21322

[45] Fassina A, Gazziero A, Zardo D, Corradin M, Aldighieri E et al. (2009) Detection of EGFR and KRAS mutations on trans-thoracic needle aspiration of lung nodules by high resolution melting analysis. J Clin Pathol 62: 1096-1102. doi:10.1136/jcp.2009.067587. PubMed: 19640859. 19640859

[46] Morandi L, de Biase D, Visani M, Cesari V, De Maglio G et al. (2012) Allele specific locked nucleic acid quantitative PCR (ASLNAqPCR): an accurate and cost-effective assay to diagnose and quantify KRAS and BRAF mutation. PLOS ONE 7: e36084. doi:10.1371/journal.pone.0036084. PubMed: 22558339. 22558339 PMC3340405

[47] Patel RK, Jain M (2012) NGS QC Toolkit: a toolkit for quality control of next generation sequencing data. PLOS ONE 7: e30619. doi:10.1371/journal.pone.0030619. PubMed: 22312429. 22312429 PMC3270013

[48] Campbell PJ, Pleasance ED, Stephens PJ, Dicks E, Rance R et al. (2008) Subclonal phylogenetic structures in cancer revealed by ultra-deep sequencing. Proc Natl Acad Sci U S A 105: 13081-13086. doi:10.1073/pnas.0801523105. PubMed: 18723673. 18723673 PMC2529122

[49] Williams C, Pontén F, Moberg C, Söderkvist P, Uhlén M et al. (1999) A high frequency of sequence alterations is due to formalin fixation of archival specimens. Am J Pathol 155: 1467-1471. doi:10.1016/S0002-9440(10)65461-2. PubMed: 10550302. 10550302 PMC1866966

[50] Hadd AG, Houghton J, Choudhary A, Sah S, Chen L et al. (2013) Targeted, high-depth, next-generation sequencing of cancer genes in formalin-fixed, paraffin-embedded and fine-needle aspiration tumor specimens. J Mol Diagn 15: 234-247. doi:10.1016/j.jmoldx.2012.11.006. PubMed: 23321017. 23321017

[51] Chin EL, da Silva C, Hegde M (2013) Assessment of clinical analytical sensitivity and specificity of next-generation sequencing for detection of simple and complex mutations. BMC Genet 14: 6. doi:10.1186/1471-2164-14-S3-S6. PubMed: 23418865. 23418865 PMC3599218

[52] Rosell R, Molina MA, Serrano MJ (2012) EGFR mutations in circulating tumour DNA. Lancet Oncol 13: 971-973. doi:10.1016/S1470-2045(12)70369-8. PubMed: 23026821. 23026821

[53] Tuononen K, Mäki-Nevala S, Sarhadi VK, Wirtanen A, Rönty M et al. (2013) Comparison of targeted next-generation sequencing (NGS) and real-time PCR in the detection of EGFR, KRAS, and BRAF mutations on formalin-fixed, paraffin-embedded tumor material of non-small cell lung carcinoma-superiority of NGS. Genes Chromosomes Cancer 52: 503-511. doi:10.1002/gcc.22047. PubMed: 23362162. 23362162

[54] Moskalev EA, Stöhr R, Rieker R, Hebele S, Fuchs F et al. (2013) Increased detection rates of EGFR and KRAS mutations in NSCLC specimens with low tumour cell content by 454 deep sequencing. Virchows Arch 462: 409-419. doi:10.1007/s00428-013-1376-6. PubMed: 23468066. 23468066 PMC3624006

[55] Buttitta F, Felicioni L, Del Grammastro M, Filice G, Di Lorito A et al. (2013) Effective Assessment of egfr Mutation Status in Bronchoalveolar Lavage and Pleural Fluids by Next-Generation Sequencing. Clin Cancer Res 19: 691-698. doi:10.1158/1078-0432.CCR-12-1958. PubMed: 23243218. 23243218

[56] Marchetti A, Del Grammastro M, Filice G, Felicioni L, Rossi G et al. (2012) Complex mutations & subpopulations of deletions at exon 19 of EGFR in NSCLC revealed by next generation sequencing: potential clinical implications. PLOS ONE 7: e42164. doi:10.1371/journal.pone.0042164. PubMed: 22848739. 22848739 PMC3407088

[57] Querings S, Altmüller J, Ansén S, Zander T, Seidel D et al. (2011) Benchmarking of mutation diagnostics in clinical lung cancer specimens. PLOS ONE 6: e19601. doi:10.1371/journal.pone.0019601. PubMed: 21573178. 21573178 PMC3088700

[58] Oshita F, Matsukuma S, Yoshihara M, Sakuma Y, Ohgane N et al. (2006) Novel heteroduplex method using small cytology specimens with a remarkably high success rate for analysing EGFR gene mutations with a significant correlation to gefitinib efficacy in non-small-cell lung cancer. Br J Cancer 95: 1070-1075. doi:10.1038/sj.bjc.6603396. PubMed: 17047654. 17047654 PMC2360725

[59] Lozano MD, Zulueta JJ, Echeveste JI, Gúrpide A, Seijo LM et al. (2011) Assessment of epidermal growth factor receptor and K-ras mutation status in cytological stained smears of non-small cell lung cancer patients: correlation with clinical outcomes. Oncologist 16: 877-885. doi:10.1634/theoncologist.2010-0155. PubMed: 21572125. 21572125 PMC3228207

[60] Rekhtman N, Brandt SM, Sigel CS, Friedlander MA, Riely GJ et al. (2011) Suitability of thoracic cytology for new therapeutic paradigms in non-small cell lung carcinoma: high accuracy of tumor subtyping and feasibility of EGFR and KRAS molecular testing. J Thorac Oncol 6: 451-458. doi:10.1097/JTO.0b013e31820517a3. PubMed: 21266922. 21266922

[61] da Cunha Santos G, Saieg MA, Geddie W, Leighl N (2011) EGFR gene status in cytological samples of nonsmall cell lung carcinoma: controversies and opportunities. Cancer Cytopathol 119: 80-91. doi:10.1002/cncy.20150. PubMed: 21400669. 21400669

[62] Na II, Kang HJ, Cho SY, Koh JS, Lee JK et al. (2007) EGFR mutations and human papillomavirus in squamous cell carcinoma of tongue and tonsil. Eur J Cancer 43: 520-526. doi:10.1016/j.ejca.2006.09.025. PubMed: 17224267. 17224267

[63] Na II, Rho JK, Choi YJ, Kim CH, Park JH et al. (2007) The survival outcomes of patients with resected non-small cell lung cancer differ according to EGFR mutations and the P21 expression. Lung Cancer 57: 96-102. doi:10.1016/j.lungcan.2007.01.027. PubMed: 17337084. 17337084

[64] Kotoula V, Sozopoulos E, Litsiou H, Fanourakis G, Koletsa T et al. (2009) Mutational analysis of the BRAF, RAS and EGFR genes in human adrenocortical carcinomas. Endocr Relat Cancer 16: 565-572. doi:10.1677/ERC-08-0101. PubMed: 19190079. 19190079

[65] Metzger B, Chambeau L, Begon DY, Faber C, Kayser J et al. (2011) The human epidermal growth factor receptor (EGFR) gene in European patients with advanced colorectal cancer harbors infrequent mutations in its tyrosine kinase domain. BMC Med Genet 12: 144. doi:10.1186/1471-2350-12-144. PubMed: 22026926. 22026926 PMC3215960

[66] Chang YL, Wu CT, Shih JY, Lee YC (2011) Unique p53 and epidermal growth factor receptor gene mutation status in 46 pulmonary lymphoepithelioma-like carcinomas. Cancer Sci 102: 282-287. doi:10.1111/j.1349-7006.2010.01768.x. PubMed: 21070477. 21070477

[67] Akbari M, Hansen MD, Halgunset J, Skorpen F, Krokan HE (2005) Low copy number DNA template can render polymerase chain reaction error prone in a sequence-dependent manner. J Mol Diagn 7: 36-39. doi:10.1016/S1525-1578(10)60006-2. PubMed: 15681472. 15681472 PMC1867510

[68] Altimari A, de Biase D, De Maglio G, Gruppioni E, Capizzi E et al. (2013) 454 next generation-sequencing outperforms allele-specific PCR, Sanger sequencing, and pyrosequencing for routine KRAS mutation analysis of formalin-fixed, paraffin-embedded samples. OncoTargets and Therapy 6: 1057-1064. PubMed: 23950653. 23950653 10.2147/OTT.S42369 PMC3741083

